# Studying the Ring-Opening Polymerization of 1,5-Dioxepan-2-one with Organocatalysts

**DOI:** 10.3390/polym11101642

**Published:** 2019-10-10

**Authors:** Jinbao Xu, Yang Chen, Wenhao Xiao, Jie Zhang, Minglu Bu, Xiaoqing Zhang, Caihong Lei

**Affiliations:** Guangdong Provincial Key Laboratory of Functional Soft Condensed Matter, School of Materials and Energy, Guangdong University of Technology, Guangzhou 510006, China; 15914486750@163.com (Y.C.); xujunbao001@163.com (W.X.); zhangjiez2019@163.com (J.Z.); buminglu@outlook.com (M.B.); zhangxq@gdut.edu.cn (X.Z.)

**Keywords:** 1,5-dioxepan-2-one, *t*-BuP_4_, DBU, TBD, organocatalyst

## Abstract

Three different organocatalysts, namely, 1-*tert*-butyl-4,4,4-tris(dimethylamino)-2,2-bis[tris (dimethylamino) phosphoranylidenamino]-2Λ^5^,4Λ^5^-catenadi(phosphazene) (*t*-BuP_4_), 1,5,7-triazabicyclo[4.4.0]dec-5-ene (TBD) and 1,8-diazabicyclo[5.4.0]undec-7-ene (DBU), have been used as 1,5-dioxepan-2-one (DXO) ring-opening polymerization (ROP) catalysts at varied reaction conditions. ^1^H NMR spectra, size exclusion chromatography (SEC) characterizations, and kinetic studies prove that the (co)polymerizations are proceeded in a controlled manner with the three organocatalysts. It is deduced that *t*-BuP_4_ and DBU catalysts are in an initiator/chain end activated ROP mechanism and TBD is in a nucleophilic ROP mechanism.

## 1. Introduction

During the last several decades, for the ring-opening polymerization (ROP) of cyclic esters, transition metal and organometallic compounds were commonly used as initiators or polymerization catalysts [1,2,3,4]. Notwithstanding the polymerization processes are very successful in producing degradable polyesters and polycarbonates, it is clear that the metallic-based compounds are environmentally sensitive, and a lack of residual metal contaminants is required in biomedical and microelectronic applications [5,6,7]. For the last two decades, organocatalysts have provided a powerful strategy for the ROP of cyclic monomers, e.g., lactones, epoxides or carbonates, as the advantages of convergence of convenience, fast rates, functional group tolerance, selectivity and easy separating. Polyesters, polycarbonates, poly(ethylene oxide)s, polyphosphoesters and polysiloxanes, etc., have been well defined with a controlled manner in molecular weight, molecular weight distribution, end groups, architecture, stereoregularity and monomers sequence by an organic acid or base catalysts [8,9,10,11,12,13,14,15,16]. Among the various organocatalysts, the most studied were phosphazenes, guanidines, and amidines. Hedrick and coworkers have prepared polycarbonates, polylactides with excellent controlling and functionality using 1,5,7-triazabicyclo[4.4.0]dec-5-ene (TBD) and 1,8-diazabicyclo[5.4.0]undec-7-ene (DBU) as the organocatalysts [17,18,19,20,21,22]. Zhao and Zhang’s group have developed a number of poly(ethylene oxide) based copolymers and polymerization methods with phosphazene catalysts [23,24,25,26]. Although organocatalysts have been widely studied for ROP, they have not been used as catalyst for ROP of cyclic 1,5-dioxepan-2-one (DXO) as far as we know.

DXO homopolymer is hydrophilic and completely amorphous with a low glass transition temperature (*T*_g_) of approximately −39 °C [27]. DXO based (co)polymers undergo hydrolysis in vitro and vivo, and is, therefore, a possible candidate in the design of bio-absorbable materials [28,29,30]. In the same time, these properties are very useful in a degradable copolymer with the hydrophobic and semi-crystalline segments, such as poly(*ε*-caprolactone) (PCL) and poly(lactide) (PLA). Copolymerization of DXO and other cyclic esters has been investigated to modify and improve the degradation properties. Statistical copolymers of DXO with lactide have shown interesting properties on degradation rates and stiffness, which could be easily altered [31].

**Scheme 1:**
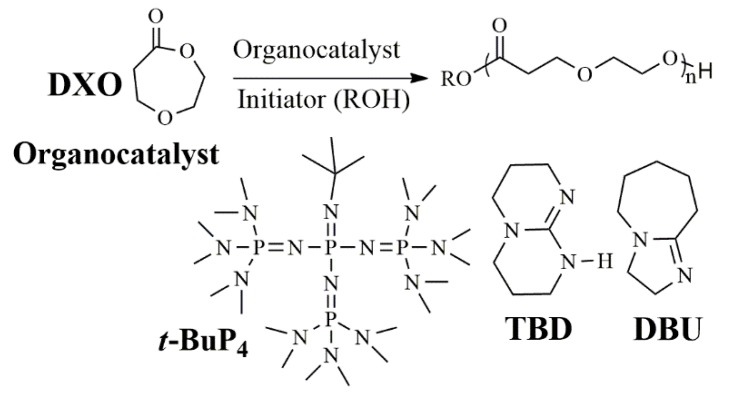
Ring-opening polymerization of 1,5-dioxepan-2-one (DXO) with *t*-BuP_4_, 1,8-diazabicyclo[5.4.0]undec-7-ene (DBU) and 1,5,7-triazabicyclo[4.4.0]dec-5-ene (TBD) as organocatalysts.

In the present work, we have studied the ROP of DXO with three different organacatalysts, 1-*tert*-butyl-4,4,4-tris(dimethylamino)-2,2-bis[tris(dimethylamino)phosphoranylidenamino]-2Λ^5^,4Λ^5^-catenadi(phosphazene) (*t*-BuP_4_, ^MeCN^p*K*a is 42.6), 1,5,7-triazabicyclo[4.4.0]dec-5-ene (TBD, ^MeCN^p*K*a is 26) and 1,8-diazabicyclo[5.4.0]undec-7-ene (DBU, ^MeCN^p*K*a is 24.3), which have verified basicity, catalytic reactivity and chemical structure (Scheme 1) [8]. We have also explored the copolymerization of DXO with CL and the initiation with poly(ethylene glycol) monomethyl ether (*m*PEG) as macroinitiator, our aim is to well know the ROP process of DXO with organocatalysts which may be useful in synthesizing biodegradable materials.

## 2. Materials and Methods 

### 2.1. Materials 

DXO was synthesized through Bayer-Villiger oxidation according to the literature, then purified by recrystallization from the dry ether, and two subsequent distillations under reduced pressure [32,33]. *ε*-Caprolactone (CL) from Aldrich (Shanghai, China) was dried over calcium hydride (CaH_2_) and distilled under reduced pressure prior to use. Ethylene glycol (EG) and benzyl alcohol (BnOH) from Sinopharm (Shanghai, China) were dried over sodium with protective nitrogen atmosphere and distilled under reduced pressure prior to use. Tetrahydrofuran (THF) and toluene (TOL) from Sinopharm (Shanghai, China) were freshly distilled from sodium/benzophenone and stored under an argon atmosphere. DBU, TBD, *t*-BuP_4_ from Aldrich (Shanghai, China) were used as received, and other reagents from Sinopharm (Shanghai, China) were also used as received.

### 2.2. Characterizations 

Proton nuclear magnetic resonance (^1^H NMR) spectra were recorded on a Bruker AV400 NMR spectrometer (Rheinstetten, Germany) by using deuterated chloroform (CDCl_3_) or benzene (C_6_D_6_) as the solvent and tetramethylsilane (TMS) as the internal standard. The apparent number average molecular weight (*M*_n_) and polydispersity index (PDI) were measured at 35 °C on a Waters size exclusion chromatography (SEC) (Milford, USA) equipped with a model 510 pump, two identical PL gel columns (5 μm, MIXED-C) and a differential refractive index detector model 410 (RI). A series of monodisperse polystyrenes were used as the standards with THF as the eluent at a flow rate of 1.0 mL/min.

### 2.3. Synthesis of PDXO Homopolymer 

A typical polymerization was performed as follows: DXO (0.58 g, 5.0 mmol, 100 equiv) and BnOH (5.0 μL, 0.05 mmol, 1.0 equiv) were added to a flask with purified THF (2 mL) at room temperature. *t*-BuP_4_ (50 μL, 0.05 mmol, 1.0 equiv in hexane) was then added with a syringe to initiate the polymerization (most of the polymerization was handled in the glove box). For kinetic study, aliquots were withdrawn in an argon flow with designed time in order to monitor monomer conversions and evolution of molar masses. The polymerization was quenched by adding 0.1 mL acetic acid, a small amount of the polymerization mixture was withdrawn and dissolved with CDCl_3_ for ^1^H NMR analysis and further diluted with THF for SEC measurement to obtain *M*_n_ and PDI. The rest polymerization mixture was diluted with THF and poured into a large excess of cold ether to precipitate the polymer which was then dried under vacuum. Polymerization of DXO with TBD, DBU and *t*-BuP_4_ as the catalyst under other conditions was carried out in a similar procedure (Table 1). ^1^H NMR (400 MHz, CDCl_3_, δ, ppm): 4.22 (–COCH_2_CH_2_OCH_2_C*H*_2_O–, 2H), 3.75 (–COCH_2_CH_2_OC*H*_2_CH_2_O–, 2H), 3.65 (–COCH_2_C*H*_2_OCH_2_CH_2_O–, 2H), 2.61 (–COC*H*_2_CH_2_OCH_2_CH_2_O–, 2H).

### 2.4. Synthesis of Copolymer

A typical procedure for copolymerization was performed as follows: DXO (0.58 g, 5.0 mmol, 50 equiv), CL (0.57 g, 5.0 mmol, 50 equiv) and BnOH (5.0 µL, 0.05 mmol, 1.0 equiv) were added to a flask with THF at room temperature in glove box. *t*-BuP_4_ (50 μL, 0.05 mmol, 1.0 equiv in hexane) was then added with a syringe to initiate the polymerization. The polymerization was quenched by adding 0.1 mL acetic acid and diluted with THF, which was then poured into a large excess of cold ether to precipitate the copolymer. The obtained copolymer was then dried under vacuum for further analysis. Copolymerization of *m*PEG and DXO was proceeded in a similar manner. ^1^H NMR (400 MHz, CDCl_3_, *δ*, ppm): 4.22 (–COCH_2_CH_2_OCH_2_C*H*_2_O–, 2H), 4.06 (–COCH_2_(CH_2_)_3_C*H*_2_O–, 2H), 3.75 (–COCH_2_CH_2_OCH_2_C*H*_2_O–, 2H), 3.65 (–COCH_2_C*H*_2_OCH_2_CH_2_O–, 2H), 2.61 (–COC*H*_2_CH_2_OCH_2_CH_2_O–, 2H), 2.25 (–COC*H*_2_(CH_2_)_3_CH_2_O–, 2H), 1.66 (–COCH_2_C*H*_2_CH_2_C*H*_2_CH_2_O–, 4H), 1.33 (–COCH_2_CH_2_C*H*_2_CH_2_CH_2_ O–, 2H).

## 3. Results and Discussion

### 3.1. Polymerization 

Polymerization of DXO was first proceeded with benzyl alcohol as the initiator because the incorporation of benzyl-ester end group could be easily detected by ^1^H NMR measurement. Table 1 presents the results of DBU, TBD and *t*-BuP_4_ catalyzed ROP of DXO at varied reaction conditions. The *M*_n_ of PDXO increases with the increasing of monomer/initiator ratio indicates that the polymerization is in a controlled manner, and we can prepare the polymers with the molecular weight as designed. The PDI for all the prepared polymers are narrow, and it becomes a little wider with the catalyst basicity increasing. Reaction time for reaching the designed *M*_n_ of these organocatalysts is much lower compared with other DXO ROP catalysts, and it decreases with the organocatalysts basicity increasing [34,35]. The conversion can reach 99% even proceeding the polymerization at −20 °C indicates that the reactivity of *t*-BuP_4_ catalyzed DXO polymerization is so high. When we raise the temperature to 60 °C, there is some interesting phenomenon for all the three catalysts. We just obtained gel after the designed reaction time, and this will be discussed below.

**Figure 1:**
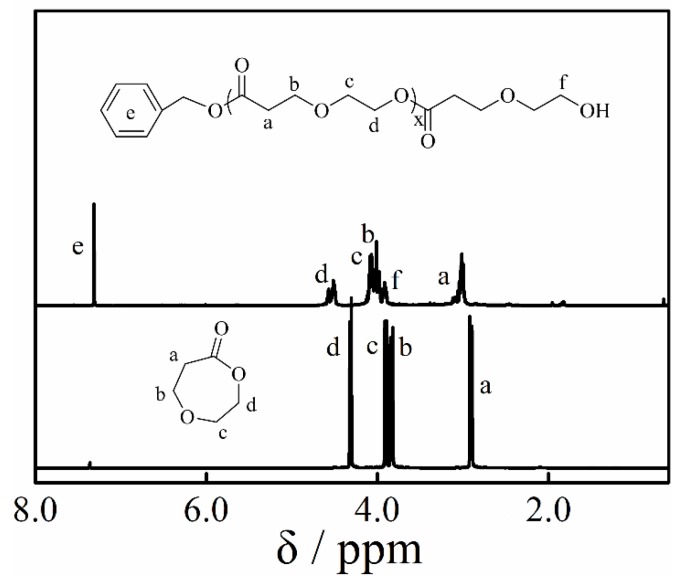
^1^H NMR spectra for DXO and PDXO (Table 1, H1).

Figure 1 shows the ^1^H NMR spectra of DXO and PDXO with *t*-BuP_4_ as the catalyst. The signals, due to the protons of DXO, PDXO main chain along with minor signals at 7.34, 5.20 ppm, due to the phenyl protons of BnO– and the methylene protons adjacent to the ester linkage for BnOH moiety of the initiator are observed. In addition, the peak, due to the methylene protons being adjacent to the *ω*-chain end of the hydroxyl group, is clearly observed at 3.70 ppm, and the peak area is comparable to that of the methylene protons of BnOH, indicating good chain-end fidelity. Moreover, the number average molecular weight of the polymer estimated from ^1^H NMR fairly agrees with that calculated from the monomer/initiator ratio and the monomer conversion (Table 1). These results indicate that polymers have the expected structure with a *ω*-chain-end hydroxyl group and *α*-chain-end benzyl group, providing one way to synthesize telechelic PDXO and PDXO-based block copolymers. Similar results could also be concluded from TBD and DBU catalyzed ROP of DXO.

### 3.2. Ether Bond Fragmentation

**Figure 2:**
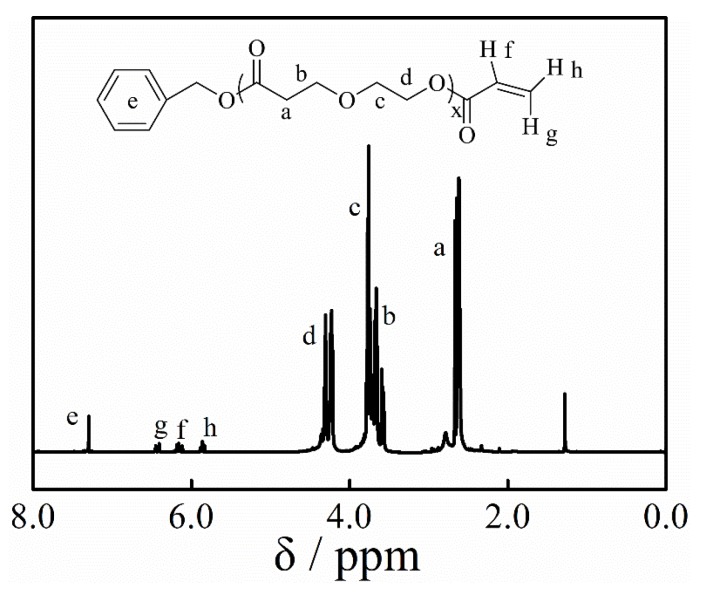
^1^H NMR spectrum of PDXO catalyzed with *t*-BuP_4_ in bulk at 60 °C (2 h) (Table 1, H6).

**Scheme 2:**
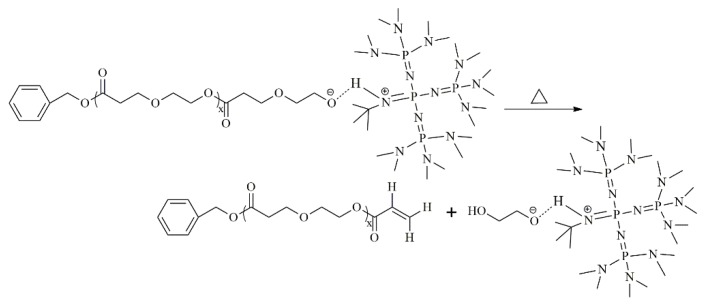
Suggested ether bond fragmentation pathway during organo-catalytic DXO polymerization.

Figure 2 shows ^1^H NMR spectrum of PDXO synthesized in bulk at 60 °C with *t*-BuP_4_ as the catalyst (Table 1, H6). The aliquot sample was taken out of reaction flask after 2 h and quenched for ^1^H NMR analysis. In addition to the signals for PDXO main chain, three new signals appear at 5.8, 6.2, 6.5 ppm which could be attributed to the three protons of double bonds, respectively. Albertsson et al. have reported this phenomenon in stannous 2-ethylhexanoate (Sn(Oct)_2_) catalyzed ROP of DXO with a temperature high than 120 °C and the unsaturated chain-end were deduced from the ether bond fragmentation [36]. However, in our experiments, the ether bond fragmentation proceeded only at 60 °C when using the three organocatalysts, and the resulted double bonds reacted spontaneously to form crosslinks between the polymer chains producing a gel after 6, 16 and 24 h, respectively. We speculate that the organocatalysts with high basicity could accelerate the ether bond fragment, and a suggested fragment pathway is shown in Scheme 2. The spontaneous reaction of unsaturated double bonds induced during thermal fragmentation of ether bonds may provide one new way for synthesizing degradable hydrogels.

### 3.3. Kinetic Study

**Figure 3:**
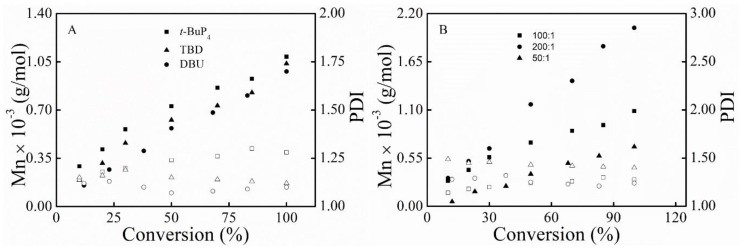
(**A**) *M*_n_ (solid) and PDI (hollow) versus monomer conversion for the ROP of DXO with *t*-BuP_4_ (square), TBD (triangle) and DBU (circle) as catalysts ([M]_0_/[I]_0_ = 100/1); (**B**) *M*_n_ (solid) and PDI (hollow) versus monomer conversion for different monomer to initiator ratio with *t*-BuP_4_ as catalyst.

To further confirm the controlled manner of organo-catalyzed ROP of DXO, we analyzed the *M*_n_ and PDI of the resulting PDXO as a function of the monomer conversion. The plot of *M*_n_ versus conversion is almost linear up to a high monomer conversion suggesting that the depletion of monomer in the reaction system is constant during the polymerization process (Figure 3A). The PDI of PDXO remains constant narrow throughout the polymerization, indicating that the transesterification effect is faint for the three organacatalysts. In addition, *M*_n_ increases to higher value with the increase in monomer conversion and the final *M*_n_ becomes higher with higher monomer to initiator ratio while maintaining narrow PDI with *t*-BuP_4_ as the catalyst (Figure 3B). These results clearly indicate that the organo-catalyzed ROP of DXO is in a controlled manner. 

**Figure 4:**
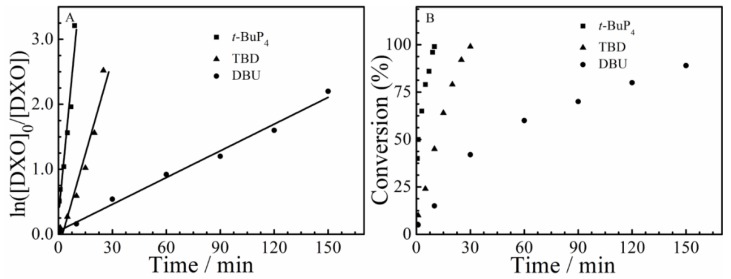
(**A**) Kinetic plots ([DXO]_0_/[I]_0_ = 50/1) and (**B**) dependence of monomer conversion on reaction time for the ROP of DXO with *t*-BuP_4_ (square), TBD (triangle) and DBU (circle) as catalysts.

Kinetics experiments were carried out to verify the kinetic order throughout the polymerization process. As shown in Figure 4A, the first-order relationship between ln([DXO]_0_/[DXO]) and the reaction time with DBU, TBD and *t*-BuP_4_ as the ROP catalysts of DXO is observed. When TBD is used as a catalyst, the conversion of DXO reaches a level of more than 75% within 20 min, and the conversion is just 38% with DBU as the catalyst. Nevertheless, the conversion of DXO reaches a level of more than 99% within 10 min when *t*-BuP_4_ is used as the catalyst. We speculate that the highest catalytic activity for *t*-BuP_4_ is attributed to its super basicity.

### 3.4. Copolymerization and Macroinitiator Initiation

**Figure 5:**
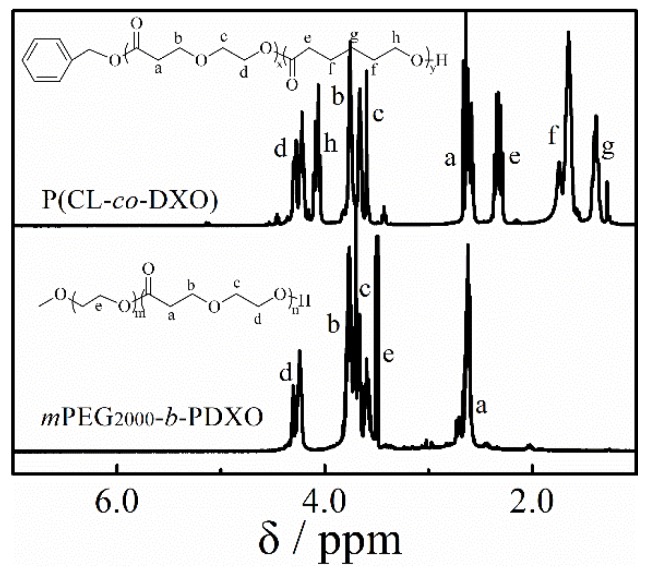
^1^H NMR spectra of P(CL-*co*-DXO) and *m*PEG_2000_-*b*-PDXO copolymers with *t*-BuP_4_ as a catalyst.

Copolymerization is the most-used route to modify and improve the properties of polymers, we proceeded the copolymerization of DXO/CL, and *m*PEG (*M*_n_ = 2000) was also used as the macroinitiator with the three catalysts. From Table 2 we can see that *m*PEG with one chain-end hydroxyl could initiate the ROP of DXO successfully; thus, a new kind of amphiphilic copolymers could be obtained. The *M*_n_ of the copolymers are as designed, the PDI are narrow, and the monomer molar ratio in the final copolymers are consistent with the initial monomers feeding calculated from ^1^H NMR (Figure 5). These results deeply demonstrate that the organo-catalyzed ROP of DXO is in a controlled manner.

### 3.5. Proposed Mechanism

**Figure 6:**
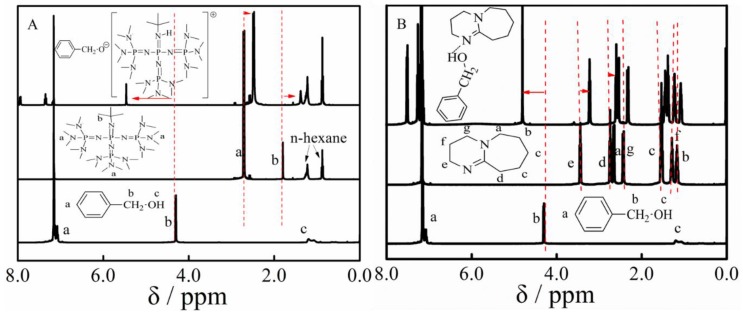
^1^H NMR spectra of (**A**) BnOH, *t*-BuP_4_ and their 1:1 complex in C_6_D_6_, (**B**) BnOH, DBU and their 1:1 complex in C_6_D_6_.

**Scheme 3:**
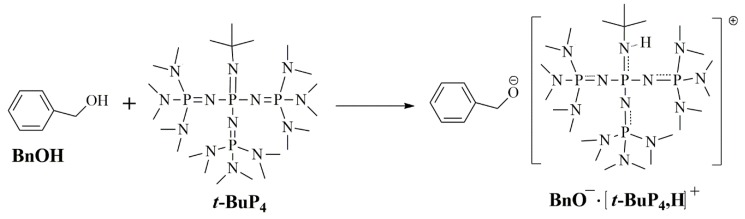
Generation of B_n_O^−^∙[*t*-BuP_4_,H]^+^ as the initiating species for the ROP of DXO.

We finally focused on the mechanism of these polymerizations, and ^1^H NMR was used to explore the possible interaction of the components in the polymerization system. Hedrick and coworkers have also used NMR to elucidate the mechanism of ROP with organocatalysts, and they indicated that MTBD form hydrogen bonds to the alcohol of an initiator [20]. Figure 6A shows the ^1^H NMR spectra of BnOH, *t*-BuP_4_ and their 1:1 complex in C_6_D_6_. For the mixture, the downfield shift of the peaks for the methylene protons of benzyl alcohol is observed from 4.3 to 5.4 ppm, while the upfield shift for *tert*-butyl protons of *t*-BuP_4_ is observed from 1.80 to 1.36 ppm and from 2.74 to 2.48 ppm for methyl protons. These results indicate that BnOH is deprotonated by *t*-BuP_4_ to form BnO^−^∙[*t*-BuP_4,_H]^+^ as shown in Scheme 3, which act as the initiating activation center in the ROP process of DXO. ROP of DXO with *t*-BuP_4_ catalyst then occurs through an initiator/chain-end polymerization mechanism by activation initiator or chain-end hydroxyls. Figure 6B shows the ^1^H NMR spectra of BnOH, DBU and their 1:1 complex in C_6_D_6_ and we conclude the similar results as in *t*-BuP_4_.

**Figure 7:**
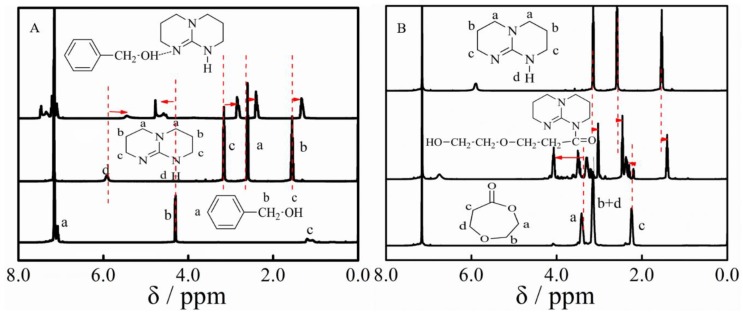
^1^H NMR spectra of (**A**) BnOH, TBD and their 1:1 complex in C_6_D_6_; (**B**) DXO, TBD and their 1:1 complex in C_6_D_6._

**Scheme 4:**
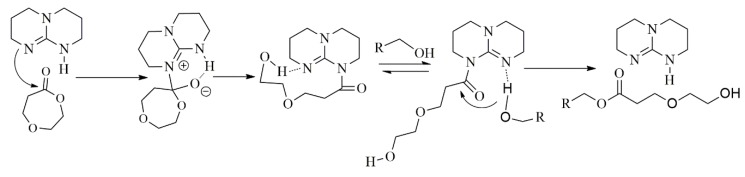
Proposed nucleophilic mechanism for the ROP of DXO with TBD as the catalyst.

Figure 7A shows the ^1^H NMR spectra of BnOH, TBD and their 1:1 complex in C_6_D_6_, and, in this condition, we conclude the similar results as in *t*-BuP_4_ and DBU. TBD and DBU have comparable basicity in MeCN; thus, the large difference in catalytic activity observed for TBD and DBU implies that the thermodynamic basicity is not the sole criterion for the ROP of DXO with these two catalysts. According to the previous publications, the role of TBD in ROP may be bifunctional, and it can also act as a nucleophile [21,37]. Figure 7B shows the ^1^H NMR spectra of DXO, TBD and their 1:1 complex in C_6_D_6_. There is quite a difference between DXO and TBD/DXO mixture. TBD could catalyze the ring-opening of DXO, and the formed hydroxyls end should act as the initiator center just as in the catalytic system of DBU and *t*-BuP_4_. These results are consistent with that the catalytic activity of TBD is much higher than DBU, and we speculate a possible nucleophilic mechanism for the ROP of DXO with TBD as the catalyst (Scheme 4). 

## 4. Conclusions

1-*tert*-butyl-4,4,4-tris(dimethylamino)-2,2-bis[tris(dimethylamino) phosphoranylidenamino]-2Λ^5^,4Λ^5^-catenadi(phosphazene) (*t*-BuP_4_), 1,5,7-triazabicyclo[4.4.0]dec-5-ene (TBD) and 1,8-diazabicyclo[5.4.0]undec-7-ene (DBU), were used as the 1,5-dioxepan-2-one (DXO) ring-opening polymerization (ROP) catalysts at varied reaction conditions. Both ^1^H NMR spectra and kinetic studies prove that the polymerization was proceeded in a controlled manner. We have also synthesized the copolymers of DXO with CL and *m*PEG by the above catalysts. Among them, *t*-BuP_4_ shows the highest catalytic behavior and DBU plays the lowest one, which is attributed to the much higher basicity of *t*-BuP_4_ than DBU. It is demonstrated that *t*-BuP_4_ and DBU proceed the ROP of DXO in an initiator/chain-end mechanism and TBD is in a nucleophilic mechanism, which can also explain the higher catalytic activity of TBD than DBU while with comparable basicity. The organocatalyzed ROP of DXO may provide one useful way for preparing PDXO based biodegradable materials and the application of polyurethane with PDXO as the soft segments are under investigation.

## Figures and Tables

**Table 1:** Characterization data of ring-opening polymerization (ROP) of DXO with three organocatalysts at various conditions ^a^.

Samples	Cat.	*f* ^b^	*T* (°C)	Time (min)	Conv. (%) ^c^	*M*_n,NMR_’ × 10^−4^ (g/mol) ^c^	*M*_n,theo_’ × 10^−^^4^ (g/mol) ^d^	*M*_n,SEC_ × 10^−4^ (g/mol) ^e^	PDI ^e^
H1	*t*-BuP_4_	50	25	5	95	0.55	0.56	0.60	1.24
H2	*t*-BuP_4_	100	25	15	96	1.10	1.17	1.08	1.28
H3	*t*-BuP_4_	200	25	120	>99	1.84	2.32	2.04	1.40
H4	*t*-BuP_4_	300	25	180	>99	2.45	3.49	3.25	1.35
H5	*t*-BuP_4_	500	25	600	>99	3.41	5.81	3.92	1.42
H6	*t*-BuP_4_	100	60	480	--	--	--	gel	--
H7	*t*-BuP_4_	50	60	800	--	--	--	gel	--
H9	*t*-BuP_4_	100	40	600	>99	1.13	1.17	1.22	1.28
H10	*t*-BuP_4_	50	−20	15	>99	0.58	0.59	0.61	1.31
H11	TBD	50	25	30	>99	0.56	0.59	0.78	1.15
H12	TBD	100	25	50	>99	1.10	1.17	1.04	1.12
H13	TBD	500	25	600	>99	3.25	5.81	3.34	1.15
H14	TBD	50	60	1080	--	--	--	gel	--
H15	DBU	50	25	180	>99	0.56	0.59	0.59	1.15
H16	DBU	100	25	600	>99	1.15	1.17	0.98	1.10
H17	DBU	500	25	1200	98	4.01	5.81	2.98	1.13
H18	DBU	50	60	1440	--	--	--	gel	--

^a^ BnOH was used as initiator (H6, H7, H9, H14, H18 with EG), *T* = 25 ℃, THF as solvent, other polymerization at 60 °C was in bulk; ^b^ molar ratio of DXO to initiator; ^c^ calculated from ^1^H NMR analysis; ^d^ calculated from ([M]_0_/[I]_0_) × monomer conversion × (*M*_W_ of DXO) + (*M*_W_ of initiator); ^e^ Obtained from SEC (size exclusion chromatography) analysis. PDI, polydispersity index.

**Table 2:** Characterization data of DXO based copolymers ^a^.

Samples	Initiators	Catalysts	*f* ^b^	*M*_n_ × 10^−4^ (g/mol) ^c^	PDI ^c^
S1	*m*PEG_2000_-OH	*t*-BuP_4_	DXO	0.72	1.24
S2	*m*PEG_2000_-OH	TBD	DXO	0.70	1.14
S3	*m*PEG_2000_-OH	DBU	DXO	0.73	1.10
S4 ^c^	BnOH	*t*-BuP_4_	CL/DXO	1.03	1.37
S5	BnOH	TBD	CL/DXO	1.03	1.27
S6	BnOH	DBU	CL/DXO	1.12	1.09

^a^*T* = 25 °C, THF as solvent; ^b^ Molar ratio of monomer to initiator is 100:1, molar ratio of CL:DXO = 1:1; ^c^ Obtained from SEC.
